# An Efficient and Accurate SCF Algorithm for Block Copolymer Films and Brushes Using Adaptive Discretizations

**DOI:** 10.3390/polym16091228

**Published:** 2024-04-27

**Authors:** Le Qiao, Marios Giannakou, Friederike Schmid

**Affiliations:** Institut für Physik, Johannes Gutenberg-Universität Mainz, D55099 Mainz, Germany; mgiannak@uni-mainz.de

**Keywords:** SCF, BCP, brush, adaptive, film, polymer

## Abstract

Self-consistent field (SCF) theory serves as a robust tool for unraveling the intricate behavior exhibited by soft polymeric materials. However, the accuracy and efficiency of SCF calculations are crucially dependent on the numerical methods employed for system discretization and equation-solving. Here, we introduce a simple three dimensional SCF algorithm that uses real-space methods and adaptive discretization, offering improved accuracy and efficiency for simulating polymeric systems at surfaces. Our algorithm’s efficacy is demonstrated through simulations of two distinct polymeric systems, namely, block copolymer (BCP) films and polymer brushes. By enhancing spatial resolution in regions influenced by external forces and employing finer contour discretization at grafting chain ends, we achieve significantly more accurate results at very little additional cost, enabling the study of 3D polymeric systems that were previously computationally challenging. To facilitate the widespread use of the algorithm, we have made our 1D-3D SCF code publicly available.

## 1. Introduction

Self-assembled copolymer materials have diverse applications in both industry and daily life due to the wide and complex spectrum of possible morphological patterns into which the copolymer molecules may assemble spontaneously. Self-consistent field (SCF) theory, which employs a simplified chain representation and a mean-field approximation to predict the spatial distribution of polymer segments, has proven to be a reliable and powerful tool for predicting the equilibrium morphology of many polymeric systems [1,2,3].

The SCF calculation iteratively computes space-dependent polymer density and associated potential fields from chain statistics propagators until the self-consistent condition is satisfied [4]. The chain propagators satisfy nonlinear modified diffusion equations in the variables space and chain contour (“time”), which have to be numerically solved using spectral or real-space methods. Pure spectral methods based on, e.g., Fourier series-based spectral solutions, have the advantage that they do not require a discretization of the chain contour. Matsen and coworkers have demonstrated their capability in accurately constructing morphology phase diagrams for periodic block-copolymer melts [3,5,6,7]. However, they demand prior knowledge of morphology, assuming symmetry in the considered phase, which limits their applicability for discovering new phases [8,9,10,11]. To address these limitations, the pseudo-spectral method was introduced, which does rely on a discretization of the contour and switches between Fourier and real-space representations of the system, utilizing the Fourier representation for the evaluation of gradient terms and the real-space representation for the evaluation of nonlinear self-consistent fields [10,12]. In contrast, pure real-space methods discretize the diffusion equation within a simulation box and solve for the solution using finite difference schemes [8,13,14,15,16]. This approach is particularly advantageous for complex polymer systems characterized by symmetry breaking or non-periodic boundary conditions, but can be computational expensive in three dimensions. A comprehensive exploration of the advantages and disadvantages of these methods can be found in Ceniceros and Frederickson’s detailed review [11].

Our primary objective in this paper is to enhance the efficiency and accuracy of real-space methods, which heavily rely on the discretization of space/chain contours. The solution of modified diffusion equations in the SCF context typically employs lower-order finite difference; however, unless the discretization is very fine, the accuracy and stability of SCF calculations can suffer, especially when dealing with polymer systems containing sharp interfaces [17,18]. For instance, in simulating polymer films, in which the substrate/air interface is often represented by a Dirichlet boundary condition and surface interactions with the polymer are introduced artificially through an external potential field, numerical inaccuracies can significantly affect the calculated free energy of the film [19]. Similarly, in the context of polymer brushes [14,20], ensuring proper attachment of the grafting end to the substrate involves fixing an end segment on one space grid using a Dirac delta function as the initial condition for the modified diffusion equations [2,14,21]. Achieving convergence to an accurate SCF solution necessitates much finer contour discretization compared to free chains, demanding additional computational effort.

The trade-off between spatial discretization and computational efficiency presents a critical challenge, especially for systems requiring higher-dimensional calculations such as cylinders and spheres in thin copolymer films [19,22,23] or particle-grafted chain polymer brushes with angular or radial-dependent morphologies [21,24,25]. To address this challenge, our paper introduces a simple scheme that adaptively increases discretization in the spatial domain where external forces are present and refines the discretization in the contour domain at the grafting point. This approach is similar in spirit to other more sophisticated adaptive methods that have recently been proposed in the literature, such as the use of Oc-Tree data structures [26], polygonal meshes [27], and finite element methods [16,28]. By optimizing the spatial resolution according to the system’s composition, our approach achieves very high accuracy while keeping computational resources low compared to uniform finite-difference grid methods. In the following sections, we evaluate and demonstrate the effectiveness of our adaptive scheme with two test cases of polymeric systems, namely, block copolymer (BCP) films and polymer brushes.

## 2. Background: SCF Equations for Two Test Cases

We use the SCF theory for inhomogeneous systems of Gaussian polymers [29]. In the following, we list only the most important equations relevant for our test systems; readers may refer to the literature for the details of the derivations [30].

### 2.1. Test Case 1: Diblock Copolymer Film

We consider an incompressible melt of asymmetric AB diblock copolymer molecules with a degree of polymerization *N*, which is confined between two flat surfaces. We assume that the majority block A occupies a volume fraction *f* of each diblock copolymer chain and that both blocks share the same statistical segment length *b*. In the grand canonical ensemble, the free energy takes the following form [19]:

(1) $$\begin{array}{cc} \frac{F}{k_{b}T}= & -e^{\mu }Q+\rho_{c}\int dr\left[\chi N\phi_{A}\left(r\right)\phi_{B}\left(r\right)+\frac{1}{2}\kappa N{\left(\phi_{A}\left(r\right)+\phi_{B}\left(r\right)-1\right)}^{2}\right]\\ \; & -\rho_{c}\int dr\left[\omega_{A}\left(r\right)\phi_{A}\left(r\right)+\omega_{B}\left(r\right)\phi_{B}\left(r\right)\right]\\ \; & +\rho_{c}\int drH\left(r\right)N\left[\Lambda_{A}\phi_{A}\left(r\right)+\Lambda_{B}\phi_{B}\left(r\right)\right] \end{array}$$

where $\mu =\mu_{0}+\ln G$ is the chemical potential, $G=\rho_{c}R_{g}^{3}$ is the rescaled dimensionless copolymer density in the bulk, $\rho_{c}=n/V$ is the average molecular number density (with *n* being the total number of copolymer molecules and *V* the volume of the film), $R_{g}=\sqrt{Nb^{2}/6}$ is the radius of gyration of the noninteracting copolymer chain, and serves as the spatial length unit throughout the paper, *Q* is the partition function of a single copolymer chain in the mean field of the other chains, $\phi_{A}\left(r\right)$ and $\phi_{B}\left(r\right)$ are the local concentrations of the A and B segments at a given point r, and χ is the Flory–Huggins parameter specifying the incompatibility of the two segments. The incompressibility of the BCP melt is ensured by the inverse of the isothermal compressibility parameter κ. The last term on the right-hand side of Equation (1) describes the interaction energy with the substrate/interface, with $\Lambda_{A,B}H\left(r\right)$ being the surface field. We assume symmetric boundary wetting conditions; the surface interaction energies with A and B segments are $\Lambda_{A}$ and $\Lambda_{B}$, respectively. The surface field

(2) $$H\left(r\right)=\left\{\begin{array}{cc} \left(1+\cos(\pi z/\epsilon )\right) & 0⩽z⩽\epsilon \\ 0 & \epsilon ⩽z⩽h-\epsilon \\ \left(1+\cos(\pi (h-z)/\epsilon )\right) & h-\epsilon ⩽z⩽h \end{array}\right.$$

is applied within a depth of ϵ from the two surfaces, where *h* is the distance between the two surfaces. By finding the extremum of the free energy in Equation (1) with respect to $\phi_{A,B}\left(r\right)$, we obtain the fields experienced by the A and B segments:

(3) $$\frac{\omega_{A}\left(r\right)}{N}=\chi \phi_{B}\left(r\right)+\kappa \left[\phi_{A}\left(r\right)+\phi_{B}\left(r\right)-1\right]+\Lambda_{A}H\left(r\right),$$

(4) $$\frac{\omega_{B}\left(r\right)}{N}=\chi \phi_{A}\left(r\right)+\kappa \left[\phi_{A}\left(r\right)+\phi_{B}\left(r\right)-1\right]+\Lambda_{B}H\left(r\right).$$

The partition function for a single chain is simply $Q=\int dr\;q\left(r,s\right)q^{†}\left(r,1-s\right)$, with q(r,1−s) and $q^{†}\left(r,s\right)$ being the partial partition functions for the first Ns and last N(1−s) segments. Note that *Q* does not depend on the specific choice of *s*. The propagators q(r,s) and $q^{†}\left(r,1-s\right)$ obey the modified diffusion equation

(5) $$\frac{\partial q(r,s)}{\partial s}=\Delta q\left(r,s\right)-\omega \left(r\right)q\left(r,s\right)$$

with initial conditions q(r,0)=1 and $q^{†}\left(r,1\right)=1$. Here, Δ represents the Laplacian, r denotes the spatial coordinate in units of $R_{g}$ and 0≤s≤1 represents the chain coordinate of the coarse-grained chain segment in the units of the chain contour length $L_{c}$. The field $\omega \left(r\right)\equiv \omega_{A}\left(r\right)$ for 0≤s≤f and $\omega \left(r\right)\equiv \omega_{B}\left(r\right)$ for f<s≤1. The local concentrations of the A and B segments are simply

(6) $$\phi_{A}\left(r\right)=\frac{1}{\rho_{c}}e^{\mu }\int_{0}^{f}dsq\left(r,s\right)q^{†}\left(r,1-s\right),$$

(7) $$\phi_{B}\left(r\right)=\frac{1}{\rho_{c}}e^{\mu }\int_{f}^{1}dsq\left(r,s\right)q^{†}\left(r,1-s\right).$$

Starting with an initial guess of the field in Equation (5), we first solve the propagator q(r,s) and $q^{†}\left(r,1-s\right)$. Next, the new local concentrations $\phi_{A}\left(r\right)$ and $\phi_{B}\left(r\right)$ are obtained from Equations (6) and (7), respectively. Finally, these concentrations are used in Equations (3) and (4) to solve for the new fields and the old and new fields are mixed according to a prescription of choice [31]. This is repeated until convergence to either a metastable or an equilibrium state.

### 2.2. Test Case 2: Homopolymer Brush

We consider a monodisperse brush solution in which *n* linear homopolymer chains with a degree of polymerization N are grafted onto a flat substrate at one end. In the canonical ensemble, the free energy of such a system is provided by

(8) $$F/k_{B}T=\rho_{o}\int dr\left[\frac{1}{2}\nu \phi {\left(r\right)}^{2}+\Lambda H\left(r\right)\phi \left(r\right)-\phi \left(r\right)\omega \left(r\right)/N\right]-n\left(\ln\left(\rho_{o}Q/n\right)+1\right),$$

where ν is the excluded-volume parameter. Similar to the BCP film system, the interactions between the segments and the substrate where the chains are grafted are imposed by an external field H(r), as in Equation (2). The self-consistent equations are determined by the extremum of the free energy, leading to

(9) $$\begin{array}{cc} \omega (r)/N & =\nu \phi (r)+\Lambda H(r), \end{array}$$

(10) $$\begin{array}{cc} \phi (r) & =\frac{nN}{Q\rho_{o}}\int_{0}^{1}ds\;q\left(r,s\right)q^{†}\left(r,s\right), \end{array}$$

(11) $$\begin{array}{cc} Q & =\int dr\;q(r,1). \end{array}$$

The initial conditions for such a system are provided by

(12) $$\begin{array}{cc} q(r,0) & =1, \end{array}$$

(13) $$\begin{array}{cc} q^{†}\left(r,0\right) & =\delta (z-g_{p}), \end{array}$$

where $g_{p}$ is the grafting point and δ is the Dirac delta function.

## 3. Adaptive Discretization

We solve the diffusion equation using the semi-implicit Crank–Nicolson discretization scheme combined with the alternating direction implicit method (ADI) [32]. For simplicity, we illustrate the method for the one-dimensional diffusion equation. Provided that the discretization points are arranged on a grid, extension of the ADI to three dimensions is straightforward. In one dimension, the diffusion equation reads

(14) $$\frac{\partial q(z,s)}{\partial s}=\frac{\partial^{2}}{\partial z^{2}}q\left(z,s\right)-\omega \left(z,s\right)q\left(z,s\right),$$

which can be discretized in *s* and *z* as

(15) $$\begin{array}{cc} \frac{q_{i}^{m+1}-q_{i}^{m}}{\delta s_{m+1}} & =\left({∆}_{z}-\omega_{i}\right)\frac{q_{i}^{m}+q_{i}^{m+1}}{2}, \end{array}$$

where $q_{i}^{m}\equiv q\left(z_{i},s_{m}\right)$, with m={1,N} being the contour steps in *s* with contour step size $\delta s_{m}=s_{m}-s_{m-1}$ and $i=\{1,n_{z}\}$ being the spatial steps in *z* with spatially varying discretization $\delta z_{i}=z_{i}-z_{i-1}$. Taking the second-order approximation for the Laplacian $\Delta_{z}$ [33], we have $\Delta_{z}f_{i}/2\approx a_{i}f_{i-1}-b_{i}f_{i}+c_{i}f_{i+1}$, with $a_{i}=\frac{1}{\delta z_{i}\left(\delta z_{i}+\delta z_{i+1}\right)}$, $b_{i}=\frac{1}{\delta z_{i}\delta z_{i+1}}$, and $c_{i}=\frac{1}{\delta z_{i+1}\left(\delta z_{i}+\delta z_{i+1}\right)}$. Rearranging Equation (15), we obtain

(16) $$\begin{array}{cc} \; & -a_{i}\delta s_{m+1}q_{i-1}^{m+1}+\left(1+b_{i}\delta s_{m+1}+\frac{\omega_{i}\delta s_{m+1}}{2}\right)q_{i}^{m+1}-c_{i}\delta s_{m+1}q_{i+1}^{m+1}\\ & =a_{i}\delta s_{m+1}q_{i-1}^{m}+\left(1-b_{i}\delta s_{m+1}-\frac{\omega_{i}\delta s_{m+1}}{2}\right)q_{i}^{m}+c_{i}\delta s_{m+1}q_{i+1}^{m}. \end{array}$$

We can calculate the propagator $q_{i}^{m+1}$ at step m+1 from the propagator $q_{i}^{m}$ at step *m*. The key lies in choosing proper adaptive discretization tailored to the system, which enables gains in both computational time and accuracy.

In the thin film case, where the loss of accuracy mainly arises from the external potential added to mimic interactions with the substrate or air interface, we employ finer discretization only in the *z* direction where external force is present. We keep a uniform contour discretization where $\delta s/L_{c}=1/N$. Our approach involves adaptive discretization with a total of $n_{z}$ grids, achieved through either a cosine function [16] or a step function. The former has a form of $\delta z_{i}=\frac{L_{z}}{2}\left[\cos\left(\frac{i-1}{n_{z}}\pi \right)-\cos\left(\frac{i}{n_{z}}\pi \right)\right]$ for $i=\{1,n_{z}\}$, providing continuous discretization. The latter uses a finer $\delta z_{s}=\frac{\epsilon }{\alpha_{s}n_{z}}$ near the surface (z≤ϵ or $L_{z}-\epsilon <z\leq L_{z}$) and a coarser $\delta z_{b}=\frac{L_{z}-2\epsilon }{\left(1-2\alpha_{s}\right)n_{z}}$ for the rest. The latter provides finer discretization; here, $\alpha_{s}$ represents the fraction of grids allocated to the surface region ϵ. Throughout this work, we refer to these adaptive schemes as “cos” and “step”, respectively.

For polymer brushes, additional adaptive contour discretization turns out to be crucial. We utilize $\delta s_{m}=\left[\cos\left(\frac{m-1}{2N}\pi \right)-\cos\left(\frac{m}{2N}\pi \right)\right],\;m=\left\{1,N\right\}$ to discretize the contour. Similar to the approach for thin films, we employ two Dirichlet wall boundaries in the *z* direction. The separation $L_{z}$ is chosen to be greater than the brush height to ensure that the brush’s free end is maintained. The grafting point of the polymer is located at a distance of $g_{p}=0.05$ from the substrate. Initially, we employ a uniform grid spacing of $\delta z_{g}=0.001$ for $z\leq 2g_{p}$, utilizing $n_{g}=100$ grids to ensure fine discretization at the grafting site. Subsequently, we gradually increase the discretization by $\delta z_{i}=\delta z_{g}+\frac{L_{z}-2g_{p}}{2}\left[\cos\left(\frac{i-1}{2(n_{z}-n_{g})}\pi \right)-\cos\left(\frac{i}{2(n_{z}-n_{g})}\pi \right)\right],\;i=\left\{1,n_{z}-n_{g}\right\}$.

## 4. Results

### 4.1. Block Copolymer Film

**Figure 1:**
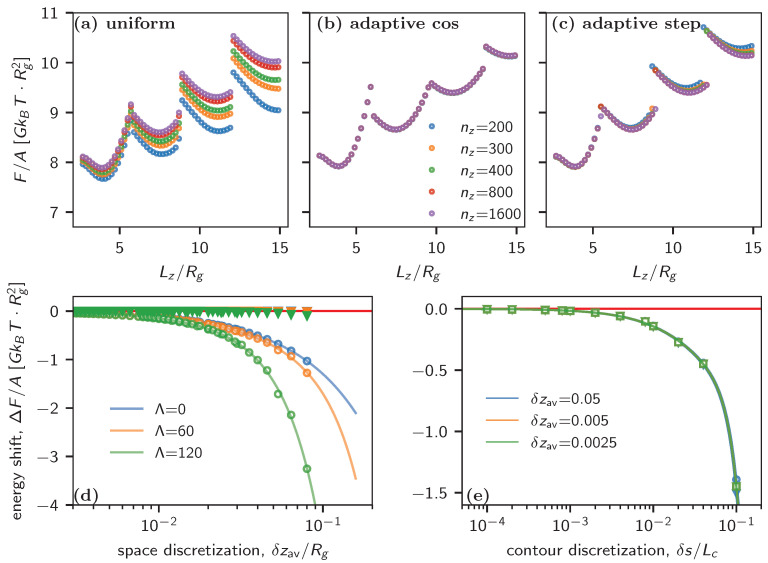
(**a**–**c**) SCF-calculated free energy F/A versus the thickness of the film $L_{z}/R_{g}$ when using the three different discretization schemes described in the text. The data are generated using f=0.5, $\mu_{0}=2.55$, χN=20, κN=25, and $\Lambda_{A}=\Lambda_{B}=60$. (**d**) Energy shift per area (ΔF/A) as a function of the averaged space discretizations $\delta z_{\mathrm{av}}$ for three different strengths of surface interactions $\Lambda_{A}=\Lambda_{B}=$ 0, 60 and 120, where ΔF=F(Δz)−F(0), with F(0) being the extrapolated value for $N_{z}=\infty$. The empty circle (∘) and solid triangle (▼) symbols represent uniform discretization and adaptive “cos” discretization, respectively, while the red horizontal line indicates ΔF=0. (**e**) Energy shift per area (ΔF/A) as a function of contour discretizations $\delta s/L_{c}$ with $\Lambda_{A}=\Lambda_{B}=60$. The empty circle (∘), square (□), and triangle (∇) indicate uniform, cos-adaptive, and step-adaptive discretization, respectively. The solid lines of different colors in (**d**,**e**) are polynomial fits of ΔF using $f\left(x\right)=ax+bx^{2}+cx^{3}$ for different surface interaction strengths and $\delta z_{\mathrm{av}}$.

We first demonstrate the effectiveness of our adaptive discretization scheme in computing the free energy of BCP films, specifically focusing on lamellar-forming BCP films (with f=0.5) as an example. Figure 1a–c illustrates the calculated free energy as a function of the film thickness using three different discretization schemes.

In the case of uniform discretization, the energy curves diverge for varying discretizations, denoted by different grid numbers. These discrepancies become more pronounced for larger thicknesses due to the coarser discretization. In contrast, our adaptive scheme consistently generates the same free energy curves regardless of the number of discretization grids used. Although a minor discrepancy arises for larger thicknesses in the step-adaptive case, the overall performance remains superior to that of the uniform case.

Figure 1d illustrates the inaccuracies versus discretization, quantified by the shift in energy from the extrapolated energy at spatial discretization δz→0. To facilitate comparison with the uniform case, the energy shift in the adaptive case is plotted versus the averaged discretization, defined by $\delta z_{\mathrm{av}}\equiv L_{z}/n_{z}$. In the uniform case, the energy shift can be fitted by a cubic polynomial, which converges to zero with increased discretization. As expected, the accuracy in the energy deteriorates more rapidly with coarser discretization when stronger surface interactions are employed. In contrast, the cos-adaptive scheme consistently produces highly accurate energy values for all tested discretizations, as is evident in the data points overlapping on the zero-error baseline. While surface interaction slightly impacts the shift in energy for larger discretizations, it is inconspicuous compared to the uniform case.

Figure 1e plots the energy shift versus $\delta s/L_{c}$ for three different discretization schemes and various $\delta z_{\mathrm{av}}$. The fact that all the curves collapse regardless of the discretization scheme and that $\delta z_{\mathrm{av}}$ indicates that adaptive discretization of the space does not require special treatment of δs. The numerical error caused by δs converges to zero when $\delta s/L_{c}<10^{-3}$ for all three cases.

Our SCF calculations for lamellar BCP films show a significant improvement in accuracy with adaptive discretization, especially when using the cos-adaptive scheme. This allows for coarser discretization, making SCF simulations more efficient and enabling the investigation of multi-layered structures in thicker films.

As a further example, we studied thin films of sphere-forming BCPs. In the bulk, prior SCF studies revealed tiny free energy differences between Hexagonally Close-Packed (HCP) and Face-Centered Cubic (FCC) packings, showing that the HCP phase is the true stable phase [7]. Here, we investigate whether this remains true for thin films. Unlike in the case of thin films with lamellar or cylindrical order, the study of sphere packings in thin films requires three-dimensional calculations and large systems, which presents a substantial computational challenge.

**Figure 2:**
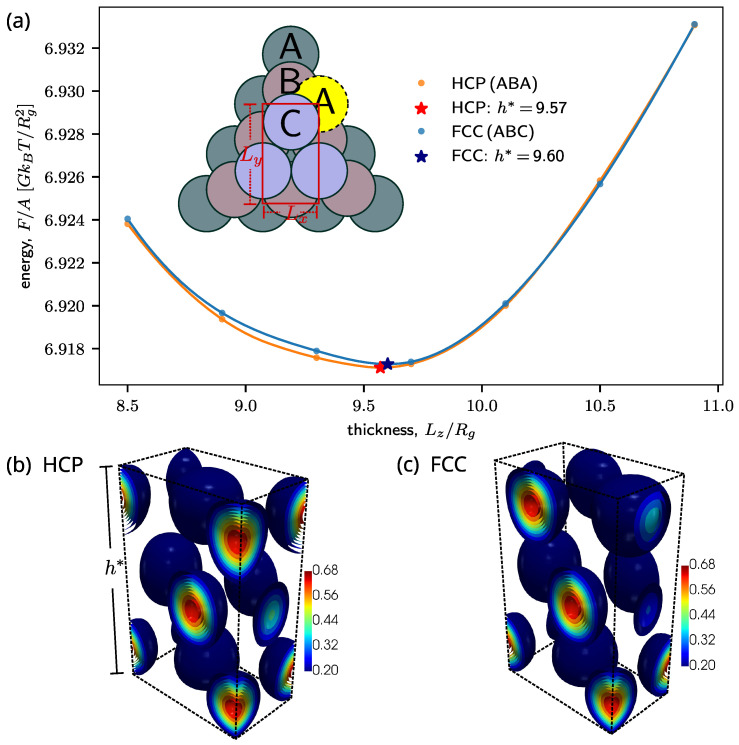
Spherical BCP film consisting of three layers. (**a**) SCF free energy per area F/A of the film as a function of film thickness $L_{z}$. The data are generated using f=0.76, $\mu_{0}=2.25$, χN=20, κN=25, $\Lambda_{A}=60$, and $\Lambda_{B}=5$. The optimum thickness, which corresponds to the minimum free energy, is marked by ★. The inset drawing shows the grid of the FCC (ABC) and HCP (ABA) packing. The green rectangle shows the selected periodic cell for the SCF calculation. (**b**,**c**): Three-dimensional contour plot of the density of the B-block calculated with the SCF for HCP and FCC packings.

Using the cos-adaptive scheme, it is possible to compute the free energy of films containing three layers of spheres (Figure 1a). The close packings of FCC and HCP correspond to ABC and ABA stackings of three layers, as illustrated by the inset drawing in Figure 2a and the SCF-calculated density plot in Figure 1b,c. The free energy curves demonstrate that the HCP phase remains the stable phase in films, i.e., it has the lower free energy. Furthermore, they show that the HCP film has a smaller equilibrium thickness (free energy minimum at $h^{*}=9.57$ $R_{g}$) than the FCC film ($h^{*}=9.60$ $R_{g}$).

### 4.2. Homopolymer Brush

Next, we consider polymer brushes. In this case, inaccuracies in SCF calculations continue to arise due to spatial discretization errors; in addition, the SCF results turn out to suffer from contour discretization errors as well. Specifically, implementing the delta function to graft the chain onto the substrate demands a much smaller δs compared to free polymer chains. Additionally, as the system approaches the strong stretching limit, which is characterized by significant chain interactions and brush thicknesses much larger than the radius of gyration of free chains, $R_{g}$, SCF calculations typically face convergence issues unless a very fine contour discretization is chosen.

**Figure 3:**
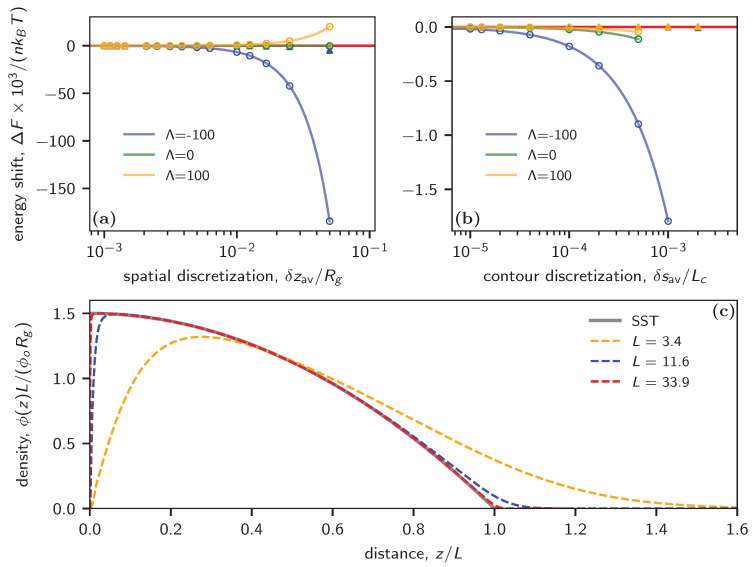
(**a**) SCF-calculated energy shift per polymer as a function of the averaged spatial discretization with attractive (Λ=−100), repulsive (Λ=100), and neutral (Λ=0) interactions with the substrate. Here, $\phi_{0}=2$ and Nν=1. (**b**) SCF-calculated free energy per polymer as a function of the contour discretization. In (**a**,**b**), the empty circles correspond to uniform discretization in space (**a**) and contour (**b**), while the filled triangles correspond to their counterparts using adaptive discretization. The red horizontal line indicates zero error ΔF=0. The solid curves in (**a**,**b**) are fits to the function $f\left(x\right)=ax+bx^{2}+cx^{3}$. (**c**) Normalized segment density $\phi \left(z\right)L/\phi_{0}R_{g}$ as a function of z/L (see text for definitions) at Λ=0. The distance is rescaled by the thickness of the brush estimated by the Strong Stretching Theory (SST).

Figure 3a plots the energy shift as a function of the averaged spatial discretization $\delta z_{\mathrm{av}}/R_{g}$ for both uniform and adaptive spatial discretizations and a fixed contour discretization of $\delta s/L_{c}=0.0001$. Similar to the previous example of the thin film, the calculations perform badly in the case of uniform discretization as δz increases. This is particularly evident in scenarios with attractive walls, where more segments are attracted to regions with external potential (the blue curve). Conversely, cos-adaptive $\delta z_{\mathrm{av}}$ consistently yields much more accurate energy calculations, as illustrated by the fact that all filled symbols align with the zero-error baseline.

In Figure 3b, we compare the energy shift relative to the average contour discretization $\delta s_{\mathrm{av}}$ for both uniform and cos-adaptive methods; we consider various wall interactions while maintaining a small uniform $\delta z/R_{g}=0.002$ throughout the analysis. For the case of uniform δs, the energy shift converges to zero at around $\delta s/L_{c}=10^{-5}$, contrasting with $10^{-3}$ in the free chain melt case. Moreover, the SCF calculation fails to converge beyond $\delta s/L_{c}⪆10^{-3}$ under uniform conditions. However, the adaptive δs scheme is able to accommodate larger discretization values while consistently maintaining significantly higher accuracy for different types of wall interactions, as shown by the empty triangles.

We further extend our calculations to the strong stretching limit. Strong Stretching Theory (SST) predicts a parabolic density profile for the polymer [34,35]. The normalized concentration can be written as

(17) $$\phi_{sst}\left(z\right)=\frac{3}{2}\phi_{o}\left(1-{\left(\frac{z}{L}\right)}^{2}\right)\frac{R_{g}}{L},$$

where $\phi_{0}=\frac{\sqrt{6}N^{1/2}n}{b\rho_{o}A}$ is the renormalization factor for the concentration and $\frac{L}{R_{g}}={\left(\frac{24N\nu }{\pi^{2}}\phi_{0}\right)}^{1/3}$ is the absolute thickness of the brush.

The SCF-calculated densities are showcased in Figure 3c. In the dilute regime, which is characterized by small *L*, the SCF captures both the depletion region near the wall and the tail at the top of the brush. As the graft density escalates, the monomer density profile in the brush approaches the prediction of the strong stretching limit. Here, the depletion width narrows, accompanied by a sharp concentration increase from zero. Despite nearing the strong stretching limit, our SCF calculations using the adaptive scheme continue to deliver accurate results. This is evident from the close alignment between the SST theory and the red dashed curve, particularly notable at L=33.9.

## 5. Conclusions

We have proposed a method for incorporating adaptive discretization schemes in real-space SCF calculations to improve both their accuracy and computational efficiency. By implementing finer discretization near surfaces, where strong polymer–substrate interactions occur, the proposed method effectively reduces numerical errors in the calculated free energy. Notably, our study shows that the cos-adaptive scheme consistently achieves superior accuracy, even with spatial discretizations larger than $\delta z_{\mathrm{av}}>0.1$, outperforming the uniform scheme by requiring discretizations that are at least ten times smaller.

To illustrate the potential of our method, we studied the morphologies of sphere-forming BCP films, focusing on the question of whether HCP or FCC stacking is more favorable. Our analysis indicates that HCP stacking is the thermodynamically stable state in thin films, at least in the example studied by us (confinement between two attractive surfaces); however, the free energy differences between HCP and FCC stacked films are very small.

When looking at polymer brushes, we again found that adaptive spatial discretization significantly enhances the accuracy of SCF calculations. In addition, adaptive discretization of the contour length parameter turns out to be essential for obtaining accurate results as well as for obtaining converged SCF solutions to begin with when the total number of discretization points is low. Choosing finer values of $\delta s_{\mathrm{av}}$ enables more subtle features to be captured, such as the depletion region near the grafting point, which are easily overlooked when using uniform discretization schemes, especially if δz is comparable to the depletion width. Importantly, our adaptive scheme alleviates the computational burden associated with brush simulations by allowing larger values of the averaged discretization parameter $\delta s_{\mathrm{av}}$ to be chosen, similar to the case of ungrafted chains in the bulk, without sacrificing accuracy. This enhancement in efficiency is particularly noteworthy in view of the fact that the costs of SCF calculations are dominated by the costs of repeatedly solving modified diffusion equations in order to obtain the propagators *q* and $q^{†}$. Furthermore, our adaptive scheme remains robust in the strong stretching limit, accommodating scenarios where inter-chain interactions exert substantial influence, resulting in brush heights $L\gg R_{g}$. While polymer brush SCF calculations in one dimension remain feasible with manageable computational costs even when using uniform discretization schemes, the integration of adaptive contour discretization and spatial discretization extends the computational capabilities, facilitating SCFT calculations in two or three dimensions for intricate morphologies [21,24,25,36,37].

In summary, our study highlights the crucial role of adaptive discretization schemes in advancing SCF calculations, delivering significant enhancements in accuracy and computational efficiency for various polymeric systems. The versatility of our simple approach is demonstrated by two illustrative examples showcasing its applicability to problems involving interfaces and external potentials. In general, the required accuracy of SCF calculations depends on the specific quantity of interest. In the present paper, we have mainly focused on the free energy, which helps to identify the true equilibrium phase from a set of competing candidate structures. In other applications, accurate prediction of the density profiles of specific chain segments may be more important.

We have introduced two types of adaptive discretization schemes, namely, adaptive spatial discretization and adaptive contour discretization. Adaptive spatial discretization is useful in all situations where density or composition profiles vary strongly within the selected regions in space. On the other hand, adaptive contour discretization is useful in situations where particularly strong variations of the propagator function q(r,s) are expected for well-defined values of *s* as is the case, for instance, with polymer brushes or copolymers close to junction points that connect different blocks. In general, it would be desirable to couple spatial discretization and contour discretization. This is because the basic equation of any SCF iteration scheme, Equation (5), has the form of a modified diffusion equation. In such cases, the contour step should not be chosen independent of the spatial discretization [38,39]. In fact, an upper bound for δs should typically scale with δz, as $\delta s∼\delta z^{1+\epsilon }$ with ϵ=2 [39]. Here, we have chosen a sufficiently small δs, i.e., smaller than the upper bound. In future work, we will explore possibilities for coupling the spatial and contour length discretizations such that the contour discretization is adjusted and becomes finer in those regions of space where the spatial discretization is finer.

It should be noted that the term “adaptive” in the present work refers to situations in which a specific inhomogeneous discretization scheme is chosen at the beginning of an SCF calculation and is not changed thereafter. In fact, we would strongly recommend not proceeding otherwise, as changing the discretization in the middle of an SCF iteration loop is likely to result in convergence problems. However, in algorithms that involve many successive SCF calculations, such as dynamic density functional (DDF) simulations, our scheme could be used to set up dynamically adaptive grids that adjust to the current state of the polymer system.

To foster broader adoption and further refinement, we have made our SCF code publicly available (see below). The code has a modular structure; it provides both adaptive real-space methods and uniform discretization pseudospectral methods, as well as, in the latter case, options to perform dynamic density functional (DDF) simulations following the DDF models used in [40,41,42]. It can be applied to arbitrary mixtures of linear multiblock copolymers, and can easily be extended to other polymer architectures as well.

## Data Availability

The code used to produce the data in the manuscript is available at https://github.com/leqiao/ADSCF.git (accessed on 24 April 2024).
